# Balancing Innovation and Safety: Frameworks and Considerations for the Governance of Dual-Use Research of Concern and Potential Pandemic Pathogens

**DOI:** 10.1089/apb.2024.0033

**Published:** 2025-06-05

**Authors:** David R. Gillum

**Affiliations:** ^1^ School for the Future of Innovation in Society, Arizona State University, Tempe, Arizona, USA.; ^2^ Compliance and Research Administration, University of Nevada, Reno, Reno, Nevada, USA.

**Keywords:** dual-use research, DURC, pathogens with enhanced pandemic potential, PEPP, biosafety, biosecurity, governance, bioethics

## Abstract

**Background:**

Governance of high-risk biological research, specifically dual-use research of concern (DURC) and pathogens with enhanced pandemic potential (PEPP), is a topic of renewed interest. This study considers the historical evolution of biosecurity policies, highlighting current challenges in balancing scientific progress with national security and public safety.

**Methods:**

A historical analysis and a literature review were conducted, examining significant events and policy developments shaping biosafety and biosecurity in the United States. The study also reviews possible frameworks for governing DURC and PEPP, assessing ethical, political, and regulatory perspectives from relevant literature.

**Results:**

Findings indicate that biosafety and biosecurity policies have historically been reactionary, responding to specific incidents rather than proactively managing risks. Despite significant policy efforts, gaps in transparency, oversight, and international collaboration persist, raising concerns about the effectiveness of governance structures. However, looking at past frameworks for managing high-risk biotechnological risks may be beneficial in establishing future governance strategies.

**Discussion:**

The study suggests the need for a balanced approach that integrates ethical, social, legal, and other considerations to ensure robust oversight of DURC and PEPP. Continuous policy evolution, informed by empirical evidence and interdisciplinary collaboration, is needed for mitigating risks associated with high-stakes biological research.

**Conclusion:**

Effective governance of DURC and PEPP requires comprehensive, interdisciplinary approaches that incorporate historical lessons, ethical considerations, and adaptive policymaking. Collaboration between policymakers, scientists, biosafety and biosecurity professionals, as well as members of the public, is required to ensure scientific innovation benefits national security and public health while minimizing risks.

## Introduction

Debate surrounding the governance of high-risk biological research is gaining momentum as Congress considers new legislation aimed at mitigating what some legislators and members of the public view as *risky research*.^1^ This article provides a historical review of significant events that have influenced biosafety and biosecurity (sometimes referred to as biological risk management or *biorisk management*) policies in the United States and explores developments involving dual-use research of concern (DURC) and pathogens with enhanced pandemic potential (PEPP). Current policies and regulations governing life sciences are discussed, along with potential frameworks for managing DURC and PEPP risks in the future. By recalling past events and policies, the article aims to provide insights into how historical approaches to biosafety and biosecurity can inform the creation of more effective strategies to address contemporary risks.

Over the past 20 years, the U.S. Government has issued biosafety and biosecurity regulations, policies, and best practices to help ensure that scientific innovation is adequately tempered to address emergent risks. Policymakers, scientists, and biosafety and biosecurity professionals are attempting to navigate the challenges of advancing knowledge while safeguarding national security and public safety. However, the tensions between fostering scientific progress and preventing the misuse of research have become increasingly pronounced.

This article begins with definitions of key terms in recent policy discussions, followed by a timeline of significant events that have shaped biosafety and biosecurity in the United States. It then explores risks and benefits of DURC and PEPP, followed by a review of the political and ethical complexities surrounding these biotechnologies. The article then examines gaps and limitations in existing governance, while advocating for a more multidisciplinary approach in developing future oversight.

## Key Definitions and Distinctions with Dual-Use Research of Concern and Pathogens with Enhanced Pandemic Potential

The concept of “dual-use research” was originally defined as research and technologies with both military and civilian applications.^2^ In 2008, the National Science Advisory Board for Biosecurity (NSABB) introduced a more specific term, “DURC.” The NSABB defined DURC as research that, based on current understanding, is reasonably anticipated to generate knowledge, products, or technologies that could be *intentionally* misapplied to threaten public health and safety, agriculture, plants, animals, the environment, or critical infrastructure.^3^ Further refining this term, the National Institutes of Health’s (NIH) Office of Intramural Research states that DURC represents a small subset of life sciences research that is distinguished by the potential for significant threats, including the misuse of knowledge, information, products, or technologies in ways that could result in broad consequences for public health, safety, agriculture, the environment, or national security.^4^ Research with PEPP involves research that can enhance the pathogenicity or transmissibility of pandemic pathogens, raising concerns about accidental release and intentional misuse.

In May 2024, the White House Office of Science and Technology Policy issued the *United States Government Policy for Oversight of DURC and PEPP*.^5^ This new policy establishes a framework to govern and oversee DURC and PEPP within the United States. The policy sets forth expectations to enhance biosecurity, promote responsible conduct of research, regulate and monitor experiments, and increase transparency and communication, especially between government agencies. The policy does not expressly call for increased funding from Congress to support biosecurity measures and research oversight.

The new policy limits oversight to specific human pathogens and toxins, such as those documented in the select agents and toxins regulated by the U.S. Department of Agriculture (USDA) and the U.S. Department of Health and Human Services.^5^ The 2024 policy also includes risk group 3 and 4 pathogens according to the *NIH Guidelines for Research Involving Recombinant or Synthetic Nucleic Acid Molecules* as well as recommendations provided in the CDC/NIH manual, *Biosafety in Microbiological and Biomedical Laboratories*.^6,7^ While research on nonhuman or plant pathogens may raise biosafety concerns, only certain human pathogens and select agents fall under the policy’s definitions. Specifically, the policy states that certain types of experiments are exempt from oversight if they do not enhance a pathogen’s transmissibility or virulence. Exempted activities include surveillance efforts like diagnostic sampling, sequencing, and virus characterization that does not involve genetic manipulation to increase pathogenic properties. Research focused on vaccine development and antiviral testing is generally not considered within its scope, provided it does not involve enhancing pathogenicity or creating PEPP.

It is important to distinguish between DURC and PEPP, as research may fall into one category without necessarily belonging to the other. For example, gene editing of certain human pathogens to study resistance mechanisms could be classified as DURC if the knowledge gained could be misused to create more virulent or dangerous pathogens. If the research does not involve pathogens capable of wide and uncontrollable spread in humans, it should not be classified as PEPP. In contrast, viral passaging in animal models bearing human receptors to identify mutations that may enable zoonotic transmission, may fall under PEPP oversight. Yet, if the research does not provide knowledge that could be misapplied to cause harm, it would not be classified as DURC. Similarly, in vaccine development, scientists regularly work with influenza strains to predict and engineer the next potential variant that could lead to widespread illness. This research could involve PEPP techniques, where viruses are modified to try and predict the next seasonal virus. Such pre-emptive research plays a critical role in global health preparedness and generally is not considered DURC.

One of the goals of the 2024 DURC/PEPP policy is to encourage public-private partnerships to leverage resources from the private sector because they are integral to ensuring that innovations move swiftly from the laboratory to practical application. Shortcomings in supply chains, such as those exposed during the COVID-19 pandemic, revealed vulnerabilities that could compromise the timely delivery of vaccines, personal protective equipment, and critical resources. Input from the private sector is, therefore, vital in addressing these challenges, streamlining production, and ensuring the resilience of global health systems.

To ensure consistency in terminology, key terms from relevant policies are provided here:

- ***DURC:*** “DURC is a subset of dual-use research defined as: ‘life sciences research that, based on current understanding, can be reasonably anticipated to provide knowledge, information, products, or technologies that could be directly misapplied to pose a significant threat with broad potential consequences to public health and safety, agricultural crops and other plants, animals, the environment, materiel, or national security’.”^8^
- ***Pathogen with Pandemic Pathogen (PPP):*** “A pathogen that is likely capable of wide and uncontrollable spread in a human population and would likely cause moderate to severe disease and/or mortality in humans.”^5^
- ***Enhanced PPP (ePPP):*** “An enhanced PPP is a PPP resulting from the enhancement of a pathogen’s transmissibility and/or virulence.”^9^
- ***PEPP:*** “A type of PPP resulting from experiments that enhance a pathogen’s transmissibility or virulence, or disrupt the effectiveness of pre-existing immunity, regardless of its progenitor agent, such that it may pose a significant threat to public health, the capacity of health systems to function, or national security. Wild-type pathogens that are circulating in or have been recovered from nature are not PEPPs but may be considered (potential pandemic pathogens) PPPs because of their pandemic potential.”^5^
- ***Gain-of-Function (GOF):*** “Any modification of a biological agent that confers new or enhanced activity.”^10^ However, the term is now considered inaccurate and unhelpful by many experts; instead, phrases like “research with pandemic potential risks” are used.
- ***Biosafety:*** “The application of practices, controls, and containment infrastructure that reduces the risk of unintentional exposure to, contamination with, release of, or harm from pathogens, toxins, and other associated biological materials.”^5^
- ***Biosecurity:*** “The application of security measures designed to prevent the loss, theft, misuse, diversion, unauthorized possession or material introduction, or intentional release of pathogens, toxins, biological materials, and related information and/or technology.”^5^
- ***Select Agents:*** “Biological agents that have the potential to pose a severe threat to animal health and safety, plant health and safety, or to the safety of animal or plant products.”^11^

Understanding the differences between these terms is necessary for developing effective governance strategies for high-risk biological research. With a common understanding of these terms, policymakers, scientists, biosafety and biosecurity professionals, and others, can better navigate complex DURC and PEPP policies.

## Significant Biosafety and Biosecurity Events

There are several important moments in the evolution of DURC and PEPP policy and related events from the 1970s to the early 2020s. These include significant discoveries, regulatory milestones, and policy discussions that have shaped the landscape of biosecurity and biosafety in the life sciences. Prior to the promulgation of the *NIH Guidelines for Research Involving Recombinant DNA Molecules,* various discoveries and policy discussions laid the groundwork for these guidelines.^6^ The NIH Guidelines established foundational biosafety protocols for genetic research and were a pivotal point in biosafety policy. The history then leads through the 21st century’s heightened concerns following biocrimes, such as the 2001 anthrax mailings, the enactment of the *Uniting and Strengthening America by Providing Appropriate Tools Required to Intercept and Obstruct Terrorism Act of 2001* to the COVID-19 pandemic. For a detailed chronological timeline of important biosafety and biosecurity events and associated references, see Supplementary Data S1.

Key developments include the establishment of the NSABB in 2004, which was formed to provide guidance on dual-use biological research. The NSABB also convened to address the controversial experiments on H5N1 influenza virus transmissibility in 2011, which sparked intense debate on the ethical implications of PEPP research.^12,13^ Subsequent policies, such as the 2013 *Framework for Guiding Funding Decisions about Research Proposals with the Potential for Generating Highly Pathogenic Avian Influenza H5N1 Viruses that are Transmissible among Mammals by Respiratory Droplets,* the 2014 *United States Government Gain-of-Function Deliberative Process and Research Funding Pause on Selected Gain-of-Function Research Involving Influenza, MERS, and SARS Viruses,* and the 2017 *Recommended Policy Guidance for Departmental Development of Review Mechanisms for Potential Pandemic Pathogen Care and Oversight*, or Potential Pandemic Pathogen Care and Oversight (P3CO) framework, reflect ongoing efforts to balance scientific advancement with biosecurity concerns.^9,10,14^

The timeline in Supplementary Data S1 also encompasses recent events, including the renewed scrutiny of PEPP research as part of the COVID-19 origins debate, detailing the evolving nature of the role of the public engagement in the scientific process. The timeline shows the efforts of the U.S. Government and scientific community to attempt to achieve a balance between advancing science, while protecting national security and public health.^15–17^

## Conceptual Frameworks to Navigate Dual-Use Research and PEPP

The dual-use nature of science, where experimentation can yield both beneficial and harmful outcomes, has long been a critical focus in biosecurity literature. Scholars have extensively reviewed and critiqued the complexities inherent in biotechnological advancements and dual-use research. The literature reviewed in this section demonstrates the need for effective governance frameworks to navigate the ethical and political challenges posed by high-risk biotechnological hazards, including DURC and experiments with PEPP. It is important to note that this literature review is not exhaustive. However, this section provides possible lenses to consider future biosafety and biosecurity governance that could be used to develop more informed and balanced governance structures.

*Historical frameworks*, such as those employed by Susan Wright^18^ and Andrew Lakoff,^19^ focus on how perceptions are shaped by social and political concerns and can be instrumental in understanding how DURC and PEPP are understood within these environments. Understanding how past political narratives can shape the definition and perception of DURC and PEPP provides valuable insights into policy responses and public attitudes. By exploring how biothreats are constructed, policymakers can sort through the hype and create governance that reflects a more balanced view of the benefits and risks of biotechnology. Similarly, frameworks that analyze past events, such as those used by W. Seth Carus^20,21^ and Glenn Cross^22^ can draw upon lessons from the past to better understand threats posed by state and nonstate actors. By understanding historical developments, stakeholders can explore the drivers that shape how state and nonstate actors choose to develop dual-use biological research and thereby potentially identify more effective strategies to prevent the misuse of biotechnology.

Frameworks that emphasize the collaborative creation [referred to as *coproduction* in the science, technology, and society, or Science and Technology Studies (STS) literature] of knowledge, such as those used by Kathleen Vogel^23^ and Sonia Ben Ouagrham-Gormley,^24^ can be used to evaluate the intersection between scientific knowledge and the social dimensions of scientific work. Applying these frameworks to DURC and PEPP research could reveal organizational and social factors that influence the governance of biotechnology. This could help to identify potential threats and gaps in current policies and develop strategies to address them.

Similarly, frameworks that assess the importance of implicit, experience-based knowledge, often referred to as *tacit knowledge*, as described by Kathleen M. Vogel,^23^ Sonia Ben Ouagrham-Gormley,^24^ Milton Leitenberg,^25,26^ and others^27,28^ can be used to describe the significance of specialized expertise in biotechnology development and how this expertise can be managed to mitigate potential threats. Policymakers can use this framework to understand the barriers and challenges in implementing biosafety and biosecurity governance. Recognizing the critical role of tacit knowledge can better inform organizational practices, training programs, and other biosafety and biosecurity controls.

*Adaptive governance* frameworks, as advocated by Michele S. Garfinkel et al.,^29^ can be useful in creating structures that balance scientific innovation with security concerns. For DURC and PEPP, this could mean developing flexible, resilient policies capable of responding to rapid technological advancements and evolving risks.

Frameworks that consider *public perception of scientific expertise*, like those used by Stephen Hilgartner^30^ and Susan Wright^18^ help to examine how scientists who take the public stage influence public policy perceptions. Effective assessment and communication strategies regarding potential biothreats are necessary for building public trust and fostering responsible conduct of research. Ensuring that governance approaches to DURC and PEPP are transparent, inclusive, and ethically sound is vital for public confidence in policy discussions.

*Analytic frameworks* and *case studies*, such as those used by Lynn Eden^31^ and Glenn Cross^32^ can be used to consider the moral and ethical implications of the governance of biological research in different settings. For DURC and PEPP, this could mean that governance frameworks should prioritize human rights, global well-being, and security, ensuring that biotechnological advances are used responsibly and align with societal values and global security norms.

Addressing the complex issues of biosafety and biosecurity in the context of DURC and PEPP will require a comprehensive and integrated approach that includes historical, social, legal, ethical, and other perspectives. An example of an interdisciplinary initiative is the World Health Organization’s *Guidance Framework on the Responsible Use of the Life Sciences: Mitigating Biorisks and Governing Dual-Use Research*, which draws on scientific, policy, and ethical perspectives to address biosecurity risks.^33^ Another example is the *Bulletin of the Atomic Scientists* report, *A Framework for Tomorrow’s Pathogen Research*, which advocates for a multidisciplinary strategy for governance that includes scientific, ethical, and policy considerations.^34^

These frameworks will help policymakers, researchers, and biosafety and biosecurity professionals address gaps in research and help to develop effective policies (see Table 1 for a typology of DURC and PEPP experiments and gaps in research). Effective governance, informed by these frameworks, will help ensure that scientific advancements contribute positively to global health and security.

**Table 1.** Typology of dual-use research of concern and pathogen with pandemic pathogen research: Science versus policy perspectives

Dimension	Science-Centric	Policy-Centric	Questions/Gaps in Research
Research Focus	Fundamental biological mechanisms, pathogen behavior, and genetics	Public health preparedness, biosecurity policies, and risk mitigation	How to better integrate scientific findings into policy decisions; balancing scientific freedom with regulatory needs
Primary Stakeholders	Scientists, research institutions, funding agencies	Policymakers, regulatory bodies, public health officials, international organizations	Ensuring all stakeholders have a voice in decision-making; understanding the impact of policies on scientific research
Goals	Advancing scientific knowledge, understanding pathogen transmission and evolution	Ensuring public safety, preventing misuse, establishing regulatory frameworks	Finding optimal ways to balance scientific progress with safety concerns
Key Activities	Laboratory experiments, gain-of-function studies, genomic sequencing	Policy formulation, regulatory oversight, risk-benefit analysis, international cooperation	Identifying effective methods for risk assessment and management; developing better communication channels between scientists and policymakers
Benefits	Insights into viral transmission, development of vaccines and therapeutics, improved disease countermeasures	Enhanced biosecurity, prevention of accidental/intentional releases, public health preparedness, international security cooperation	Measuring the long-term benefits of DURC and PPP research on public health; quantifying the impact of research restrictions
Risks	Potential for accidental release, ethical concerns over creating enhanced pathogens	Insufficient oversight, political and ethical debates, international compliance challenges	Developing comprehensive risk assessment models; understanding the global implications of local research activities
Ethical Considerations	Necessity of research vs. potential harm, informed consent, adherence to bioethics standards	Balancing humanitarian benefits with risks, transparency, global consensus on acceptable risks, robust ethical review mechanisms	Creating universally accepted ethical guidelines; evaluating the ethical implications of emerging biotechnologies
Regulatory Frameworks	Institutional biosafety committees, internal review boards, compliance with national guidelines	NSABB, P3CO Policy Framework, international treaties (e.g., Biological Weapons Convention)	Harmonizing international regulations; assessing the effectiveness of current frameworks in preventing misuse
Historical Context	Evolution of DURC policies, scientific milestones (e.g., NIH Guidelines for Recombinant DNA)	Policy responses to bioterrorism (e.g., Uniting and Strengthening America by Providing Appropriate Tools Required to Intercept and Obstruct Terrorism Act), establishment of oversight bodies (e.g., NSABB), international dialogue [e.g., Biological Weapons Convention (BWC)]	Learning from historical precedents to improve future policy; understanding how past events shape current regulations
Controversies	Gain-of-function research, ethical implications of enhancing pathogen transmissibility	Moratoriums on research funding, debates on transparency and oversight, balancing innovation with security	Addressing public concerns about DURC and PEPP research; fostering an informed and balanced public debate

This typology presents a range of DURC and PEPP research opportunities from science-focused to policy-focused dimensions. It emphasizes key components, actors, benefits, risks, and ethical issues to better understand the interactions and identify areas that require additional investigation. This framework encourages a more informed and balanced debate on the governance of high-risk scientific research. It is based on a 2015 discussion article by McNie et al. that presents a multi-dimensional framework for categorizing scientific research based on user engagement.^35^

DURC, dual-use research of concern; NSABB, National Science Advisory Board for Biosecurity; NIH National Institutes of Health; PPP, Pathogen with Pandemic Pathogen.

## Upstream, Midstream, and Downstream Considerations

**Figure 1.**
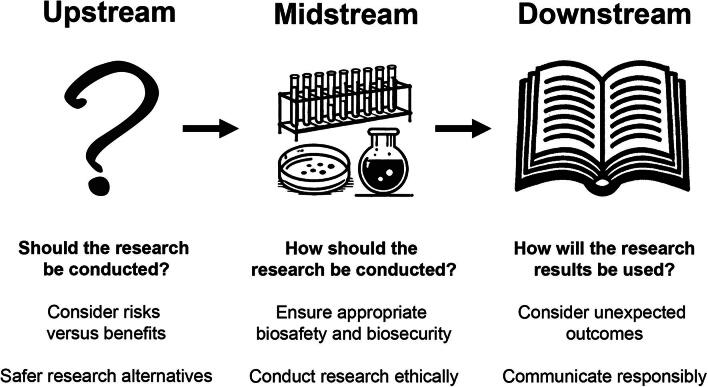
Upstream, midstream, and downstream considerations in DURC and PEPP research. DURC, dual-use research of concern; PPP, Pathogen with Pandemic Pathogen.

The challenges with DURC and PEPP research can be better understood and addressed by differentiating between the different phases of research—upstream, midstream, and downstream—each of which presents unique ethical and political concerns (see Figure 1).^36^

### Upstream Considerations: Should We Conduct the Research?

In the upstream phase, the primary ethical question is whether certain types of DURC or PEPP research should be taking place at all or if there are safer ways to conduct the research. This phase involves an evaluation of whether the anticipated benefits justify the potential risks. Ethical frameworks, such as the Nuremberg Code^37^^,^^a^ and the Belmont Report,^38^^,^^b^ provide foundational principles for making these determinations. Ethical considerations in this phase are focused on the ethical dual-use nature of such research. Some examples where scientists have worked to consider the ethical and social implications of biological research include J. Craig Venter’s work on the creation of the minimal cell—here an ethics team from the University of Pennsylvania was brought in to evaluate the proposed experiment before research commenced.^39,40^

Evans critiques policies that allow potentially dangerous research to proceed without adequately considering less risky alternatives.^41^ He advocates for the use of “alternative experiments” that could achieve similar scientific objectives with reduced risk. This aligns with broader calls from Lipsitch and Galvani, who advocate for more rigorous ethical scrutiny and comprehensive risk-benefit assessments of experiments involving PEPP. They highlight the importance of considering safer, more effective alternative research methods before engaging in high-risk biological experiments.^42^

While not all research involving PEPP is inherently dangerous, particularly when conducted under strict safety protocols and regulatory oversight, intentionally enhancing pathogenicity or transmissibility of pathogens warrants special attention and caution. Researchers have advocated for comprehensive risk-benefit analyses that acknowledge the importance of responsible conduct in high-risk research.^43,44^ As Rozell recommends, considering safer research alternatives and incorporating safe design principles into research methodologies can minimize potential harm, while advancing scientific knowledge.^45^ Similarly, Duprex et al. emphasize the importance of multiple layers of biocontainment measures to mitigate risks.^46^

### Midstream Considerations: How Should the Research be Conducted?

As research moves into the midstream phase, the ethical and political concerns shift to how the research is conducted and how to manage unexpected results. This phase requires careful attention to the methodologies employed, ensuring that research is performed under the highest standards of biosafety and biosecurity. For example, the monitoring and precautions taken during the reconstruction of the 1918 pandemic virus serve as a notable example of such midstream oversight.^47^ Comprehensive risk-benefit analyses that integrate ethical considerations are essential during this phase. Ethicists play an important role in not only framing the moral implications of the work but also providing guidance for the ethical conduct of research. The interdisciplinary collaboration of scientists, biosafety and biosecurity professionals, policymakers, ethicists, with input from the public, is essential to ensure that DURC and PEPP research is conducted in a manner that prioritizes national and public safety without stifling scientific innovation.

### Downstream Considerations: How Should Research Outcomes be Used?

The downstream phase focuses on how research outcomes are used and the potential risks, including unintended consequences, even when these outcomes are applied as originally intended. This phase requires careful consideration of the potential impacts on public health and safety, especially when research involves PEPP. In the downstream phase, the ethical dilemmas often revolve around the downstream effects of research that has already been conducted and is either ready to be published or already published. Past experiments with synthetic polio virus^48^ and mousepox virus^49^ that were published and then only later people started to think about their security risks. Sandbrink et al. discuss the dual-use potential of the outputs of viral vector research and the ethical need for safer research approaches and stricter biosecurity measures and responsible communication, arguing that current policies often fail to capture the risks associated with the transferability of insights between agents or from technologies outside of biology.^50^

An additional challenge in this phase is the inconsistency in the policies of leading professional journals regarding the identification of DURC publications. With the convergence of molecular biology, biotechnology, synthetic biology, and emerging technologies like machine learning and artificial intelligence, the oversight filter that journals provide is increasingly complex and inconsistent. In addition, the rise of unrefereed preprints and social media commentaries, which bypass traditional peer review, further complicates the dual-use risk landscape. These unregulated domains present a significant challenge in ensuring biosecurity and biosafety, as the dissemination of potentially dangerous research is no longer exclusively managed by professional journals.

Navigating the complex landscape of DURC and PEPP research requires a phased approach that integrates scientific, ethical, and policy considerations at each stage—upstream, midstream, and downstream. Enhancing transparency, fostering international collaboration, and implementing robust risk-benefit analyses are crucial steps in ensuring the responsible conduct of high-risk research. By addressing the unique ethical challenges at each phase, the scientific community can advance knowledge, while safeguarding public health and biosecurity.

## Implications of High-Risk Life Sciences Research

The scientific benefits of DURC and PEPP research are thought to include the creation of new vaccines and medical treatments, an improved understanding of pathogen behavior and enhanced emergency preparedness strategies for future pandemics.^51–53^ As an example, the *National Institute of Allergy and Infectious Diseases Pandemic Preparedness Plan* discusses the importance of proactive research on “prototype pathogens” such as Ebola and Zika, to ensure that vaccine and therapeutic development timelines are shortened during future outbreaks.^54^ By advancing our understanding of human-pathogen interactions and pandemic risks, these efforts contribute to public health preparedness, ultimately leading to more robust global responses to infectious disease outbreaks.^53^

Alternatively, the risks associated with DURC and PEPP research include an increase in accidental or intentional releases of dangerous pathogens, which could precipitate a severe public health crisis (e.g., a pandemic).^55–57^ This could lead to increased public distrust and intense debates about the ethical limits of scientific research.^58^ Also, the knowledge gained from this research, often referred to as *information hazards*, has the potential to be misused for biocrimes or political manipulation.^59^

The risk/benefit assessment for PEPP research has shifted in the post-COVID-19 era. Funding decisions must now account for evolving social and political norms. While U.S. Government policies emphasize biosafety and biosecurity, they often lack concrete examples of the benefits from the research. To improve public perception and support, clear communication is needed that describes the scientific advancements and tangible benefits of DURC and PEPP research. Maintaining high standards of scientific integrity and safety will also help create an environment that fosters innovation and builds public trust in scientific research. Examples from the COVID-19 pandemic illustrates that robust safety measures can coexist with rapid scientific advancements, demonstrating that public health priorities and scientific discovery can be mutually supportive.^60,61^

Politically, the regulation of DURC and research with PEPP has been heavily influenced by historical incidents of biosafety lapses and the increasing concern for global health threats.^42,62,63^ For example, the 2014 moratorium was a response to heightened concerns about the dual-use nature of GOF research.^10^ This was a deliberate measure instituted by the U.S. Government to address the potential risks associated with purposefully increasing the pathogenicity and transmissibility of viruses that were not previously a risk to humans.^64,65^ The moratorium was later lifted with the establishment of the 2017 P3CO framework, which provided guidelines for evaluating the risks and benefits of research with pandemic pathogens.^9^ Importantly, only four proposals have been received by HHS life sciences funding agencies that included ePPP work.^66^ This limited number may suggest differences in how scientists, biosafety and biosecurity professionals, compliance officers, and others at different institutions interpreted the definition of covered research. Another factor could be the exclusion of federally funded research from the screening process. Alternatively, it is possible that institutions were not conducting research on covered experiments. This issue warrants further study.

The possibility that COVID-19 stemmed from research-related activities has contributed to increased concern from the public regarding the safety of high-containment biological research facilities.^67–72^ Similarly, the placement of Biosafety Level 4 (BSL-4) laboratories, like the National Emerging Infectious Diseases Laboratories in Boston (USA), the National Microbiology Laboratory in Winnipeg (Canada), the Galveston National Laboratory in Texas (USA), and the Ontario Institute for Cancer Research in Etobicoke (Canada), raised safety, security, and environmental justice concerns about risks to vulnerable communities from these facilities.^73,74^ There has also been debate about the selection process for the replacement of the Plum Island Animal Disease Center in New York, USA, with the new National Bio and Agro-Defense Facility in Kansas.^75,76^

Lastly, the rapid proliferation of BSL-3 and BSL-4 laboratories in nations where standards for personnel training, audits, inspections, and overall transparency do not meet internationally accepted benchmarks, raises concerns for the accidental release or misuse of dangerous pathogens.^77^ Hence, international cooperation and independent verification mechanisms are needed to ensure that biocontainment protocols are rigorously followed at these facilities. Balancing scientific progress with national security will require careful monitoring and formal assessment mechanisms to ensure that the benefits of DURC and research with PEPP outweigh the associated risks.

## Conclusion

Addressing the complex biosafety and biosecurity issues surrounding DURC and PEPP research will require a more comprehensive, inclusive, and integrated approach. Several frameworks have been proposed in this article to help navigate the challenges posed by high-risk biotechnological hazards. These frameworks, which include *historical*, *constructivist*, *co-production*, *tacit knowledge*, and *adaptive governance models*, offer different lenses through which to consider future biosafety and biosecurity oversight. By applying these methodologies, policymakers, scientists, biosafety and biosecurity professionals, and others, including members of the public, can work to develop more informed and balanced governance structures that consider both the potential benefits and risks of DURC and PEPP research.

Continuous evaluation and improvement of existing frameworks, informed by empirical evidence and practical experience, will promote a balanced and constructive conversation. Through informed policymaking and rigorous oversight, the goals of scientific innovation, national security, and public safety can be effectively reconciled. Ultimately, the resolution of these tensions will help shape not only the boundaries of scientific exploration but also the social, ethical, legal, and related frameworks that guide it.

## Notes

^a^

*The Nuremberg Code, developed in response to the Doctors’ Trial during the Nuremberg Military Tribunals, established key principles for conducting ethical medical research, emphasizing the necessity of voluntary consent and the avoidance of unnecessary suffering and injury. These principles have had a profound impact on the life sciences, guiding the development of modern ethical standards and regulations for research.^36^*

^b^

*The Belmont Report, created by the U.S. Department of Health, Education, and Welfare in 1978, outlines ethical principles and guidelines for research involving human subjects. It focuses on three core principles: respect for persons, beneficence, and justice. These principles have been instrumental in shaping ethical research practices and regulatory frameworks in the life sciences, ensuring the protection and ethical treatment of research participants.^37^*
