# Supplementary material for: Balancing Innovation and Safety: Frameworks and Considerations for the Governance of Dual-Use Research of Concern and Potential Pandemic Pathogens
Source: Appl Biosaf. 2025 Jun 5;30(2):69–78. doi: 10.1089/apb.2024.0033 (PMC12183502; doi:10.1089/apb.2024.0033)
Supplement: Supplementary Data S1 [file apb.2024.0033_supp_datas1.pdf]

<u>Supplement A</u>: Timeline of Significant Biosafety and Biosecurity Events

October 7, 1974: NIH RAC formed. The National Institutes of Health (NIH) forms the Recombinant DNA Advisory Committee (RAC) to address the emerging ethical and safety issues related to recombinant DNA technology. (National Institutes of Health, 1974)

February 24-27, 1975: Asilomar Conference. Scientists gather at the Asilomar Conference on Recombinant DNA to discuss the potential biohazards and regulation of biotechnology, resulting in guidelines to ensure the safe use of recombinant DNA technology. (Berg et al., 1975)

July 23, 1976: NIH Guidelines for Research Involving Recombinant DNA. The National Institutes of Health (NIH) establishes guidelines for research involving recombinant DNA, setting the stage for biosafety and biosecurity protocols in genetic research. (Wivel, 2014; National Institutes of Health, 1976)

November 1983: WHO publishes first biosafety manual. The World Health Organization (WHO) publishes the first edition of its laboratory biosafety manual, providing global guidance on biosafety practices. (World Health Organization, 1983)

September 1984: First bioterrorist attack in the U.S. The Rajneeshee commune in Oregon used Salmonella to contaminate salad bars sickening over 750 people. Members of the Rajneeshee commune deliberately contaminate salad bars in The Dalles, Oregon, with Salmonella, resulting in over 750 cases of food poisoning. This incident is the first known bioterrorist attack in the United States. (Torok et al., 1997)

May 1984: First edition of CDC/NIH BMBL published. The Centers for Disease Control and Prevention (CDC) and the National Institutes of Health (NIH) publish the first edition of the "Biosafety in Microbiological and Biomedical Laboratories" (BMBL), outlining essential biosafety practices. (CDC/NIH, 1984)

June 1984: ABSA founded. The American Biological Safety Association (ABSA) is established to promote biosafety as a scientific discipline and address the growing concerns over biological safety in laboratories. (ABSA International, 1984)

April 24, 1996: Passage of first law controlling certain biological agents. Passage of the Antiterrorism and Effective Death Penalty Act of 1996 (PL 104-132), authorizing the Secretary of Health and Human Services to establish regulatory control over transfers, but not possession or use, of listed biological agents. (US Congress, 1996)

June 10, 1996: First Select Agent regulations. CDC published the "Notice of Proposed Rulemaking (NPRM) to Implement Section 511 of Public Law 104-132, 'The Antiterrorism and Effective Death Penalty Act of 1996'." (CDC, 1996).

October 24, 1996: Regulations for Transferring Select Agents. "Additional Requirements for Facilities Transferring or Receiving Select Agents" (42 CFR Part 72.6) enters into force. (US DHHS, 1996)

Fall 2001: Amerithrax attacks. In October 2001, an American Media Inc. (AMI) employee in Florida was diagnosed with inhalational anthrax, the first U.S. case in over two decades. By November 2001, 21 additional cases and 5 deaths occurred, and by December 2001, the EPA confirmed anthrax contamination at over 60 sites, including numerous postal facilities. (U.S. Government Accountability Office, 2003)

October 26, 2001: USA PATRIOT Act and bioterrorism. The USA PATRIOT Act was enacted in response to the 9/11 and Amerithrax attacks, including provisions that enhance controls over biological agents and toxins that could

be used for bioterrorism. This law builds on the then-existing Select Agent regulations by prohibiting "restricted persons" from possessing them. (USA PATRIOT Act, 2001)

June 12, 2002: Public Health Security and Bioterrorism Preparedness and Response Act of 2002. Passage of this act extended the biological select agents and toxins program and by calling for the Secretaries of Agriculture and HHS to establish a system to regulate possession of these agents by banning their transfer to or possession by any individuals or entities not registered to have them. (US Congress, 2002).

December 13, 2002: Expanded Select Agent regulations. The Federal Select Agent Program is expanded to oversee the possession, use, and transfer of biological select agents and toxins that pose a severe threat to public health and safety. The regulations were published in the Federal Register on December 13, 2002, and became effective on February 7, 2003. (US DHHS, 2002)

February 2003: Biodefense and biosecurity statement. Publications by Nature, Science, and Proceedings of the National Academy of Sciences of Statement on the Consideration of Biodefense and Biosecurity by a group of scientific journal editors, scientist-authors, government officials, and others, which among other things states that, "We recognize that on occasions an editor may conclude that the potential harm of publication outweighs the potential societal benefits. Under such circumstances, the paper should be modified, or not be published." [This followed from a meeting held on January 10, 2003, the day after a joint National Academies/Center for Strategic and International Studies conference on scientific openness and national security.] (Atlas et al., 2003).

October 18, 2003: Fink Report. Release of the National Academies' report Biotechnology Research in an Age of Terrorism, or the "Fink Report," which, among other things, identified seven classes of experiments of concern that warrant review and discussion before being conducted and recommended formation of a National Science Advisory Board for Biodefense (later enacted, with some changes, as the National Science Advisory Board for Biosecurity). (National Research Council, 2004)

March 4, 2004: Formation of the NSABB. The National Science Advisory Board for Biosecurity (NSABB) is established in the USA to advise on dual-use research issues. One of its early tasks includes reviewing the controversial reconstruction of the 1918 influenza virus. (US DHHS, 2004)

May 25, 2005: World Health Assembly resolution WHA58.29 on enhancements of lab biosafety. The World Health Assembly passes resolution WHA58.29, emphasizing the importance of laboratory biosafety and calling for enhanced measures to improve global biosafety practices. (World Health Organization, 2005)

June 30-31, 2005: Inaugural NSABB meeting. The National Science Advisory Board for Biosecurity (NSABB) holds its inaugural meeting to discuss the oversight of dual-use research and related biosecurity issues. (US DHHS, 2005)

June 2007: NSABB issues report on a proposed oversight framework for the oversight of life sciences research of concern. The NSABB publishes a report outlining a framework for the oversight of life sciences research that could be of concern for dual-use purposes. (NSABB, 2007)

October 4, 2007: HHS establishes a Trans-Federal Task Force on Optimizing Biosafety and Biocontainment Oversight. The U.S. Department of Health and Human Services (HHS) establishes a task force to enhance biosafety and biocontainment oversight across federal agencies. (US DHHS 2007)

January 9, 2009: President signs executive order (EO) 13486 was issued on strengthening lab biosecurity in the USA. President Obama signs Executive Order 13486, focusing on strengthening laboratory biosecurity measures to protect against biological threats. (US White House, 2009)

July 2, 2010: FESAP established. The President signed EO 13546, which tightened the Select Agent regulations and established FESAP. Executive Order 13546 called for a tiering of the select agent list to provide additional security for those presenting the greatest risk of misuse. It also establishes the Federal Experts Security Advisory Panel (FESAP) to optimize the security of biological select agents and toxins in the United States. (US White House, 2010)

October 13, 2010: USG issues screening framework guidance for providers of synthetic DNA. The U.S. government issues guidance for the screening of synthetic double-stranded DNA to prevent its misuse for bioterrorism or other malicious purposes. The guidance, issued by the Department of Health and Human Services (HHS), provides recommendations for commercial providers of synthetic DNA to screen orders to ensure they are not used for harmful purposes. (US DHHS, 2010)

November 2, 2010: FESAP issues Recommendations Concerning the Select Agent Program report. The Federal Experts Security Advisory Panel (FESAP) released its recommendations for improving the Select Agent Program, as called for in EO 13546. (FESAP, 2010)

Winter 2011/Spring 2012: NSABB Reviews of Controversial H5N1 Research. The NSABB meets on December 15, 2011 and March 29-30, 2012 to review research by Yoshi Kawaoka and Ron Fouchier that demonstrates that the H5N1 influenza virus can become transmissible in mammals. The NSABB recommends publication despite biosecurity concerns, leading to significant debate within the scientific community about the risks and benefits of such research. (NSABB, 2012; Herfst et al., 2012; Imperiale et al., 2018)

March 29, 2012: USG policy for oversight of life sciences dual use research of concern. The U.S. government releases the "United States Government Policy for Oversight of Life Sciences Dual Use Research of Concern (DURC)," which requires the review of U.S. government-funded research involving 15 specific agents and toxins and 7 categories of experiments. The policy mandates the development of risk mitigation plans for experiments identified as Dual Use Research of Concern. (US Government, 2012)

February 21, 2013: HHS Funding Policy for Avian Influenza. HHS of "A Framework for Guiding U.S. Department of Health and Human Services Funding Decisions about Research Proposals with the Potential for Generating Highly Pathogenic Avian Influenza H5N1 Viruses that are Transmissible among Mammals by Respiratory Droplets" (US DHHS, 2013)

August 7, 2013: H7N9 announcement. Announcement of extra oversight for H7N9 experiments (along the lines of the reviews previously issued for certain H5N1 experiments). (Jaffe et al., 2013)

September 24, 2014: Institutional Oversight Policy for DURC. The U.S. government releases an updated policy for institutional oversight of life sciences DURC, providing guidance on identifying and mitigating risks associated with research involving high-consequence pathogens and toxins, to take effect one year later. The policy aimed to ensure that institutions ensured that DURC was identified and risk mitigation measures were implemented, where applicable. [Note that this was not a revision of the 2012 Federal DURC policy, which applied to Federal funding decisions, but a new policy that governs institutions. The 2012 Federal DURC policy remained in effect.] (Office of Science and Technology Policy, 2014)

October 17, 2014: U.S. Moratorium on GOF Research. The NIH imposes a moratorium on gain-of-function (GOF) research involving PPPs, including influenza, SARS-CoV, and MERS-CoV, due to biosecurity and biosafety concerns following a series of biosafety lapses at US government laboratories. (US Government, 2014; Lipsitch and Ingelsby, 2014)

December 2014: Report of the FESAP issued. The Federal Experts Security Advisory Panel (FESAP) issued a report titled "Recommendations for the Evaluation and Oversight of Gain-of-Function Research." The report provides comprehensive recommendations for strengthening the oversight and management of gain-of-function research involving potentially pandemic pathogens, emphasizing the need for enhanced biosafety and biosecurity measures. (FESAP, 2014)

December 15-16, 2014: GOF research symposium. The U.S. National Academy of Sciences holds symposium "Gain-of-Function Research: A Symposium" (National Academies of Science, 2014)

April 13, 2015: National Academies Report of Gain-of-Function Research. The National Academies Press publishes "Potential Risks and Benefits of Gain-of-Function Research: Summary of a Workshop," discussing the ethical and practical implications of GOF research with pathogens of pandemic potential. (Sharples et al., 2015)

May 2015: NSABB issues a framework for conducting risk and benefit assessment of gain-of-function research. The NSABB publishes a framework for assessing the risks and benefits of gain-of-function research involving potentially pandemic pathogens. (NSABB, 2015)

March 10-11, 2016: Second symposium on GOF. The National Academies holds a symposium "Gain-of-Function—The Second Symposium. (National Academies of Sciences, 2016)

April 2016: Gryphon Scientific Publication. Gryphon Scientific publishes Risk and Benefit Analysis of Gain of Function Research, Final Report-April 2016. (Gryphon Scientific, 2016)

May 2016: NSABB issues report on recommendations for the evaluation and oversight of proposed gain-of-function research. The NSABB publishes recommendations for the evaluation and oversight of gain-of-function research, emphasizing the need for robust risk assessment and management strategies. (NSABB, 2016)

June 20, 2016: Second symposium on GOF: Report. A publication of National Academies of Sciences, Engineering, and Medicine regarding their 2nd workshop. (National Academies of Sciences, 2016).

January 9, 2017: P3CO policy guidance. The OSTP releases the "Recommended Policy Guidance for Departmental Development of Review Mechanisms for Potential Pandemic Pathogen Care and Oversight" (P3CO), establishing additional review processes for GOF research involving PPPs. (Office of Science and Technology Policy, 2017)

December 19, 2017: Implementation of P3CO Framework. HHS releases "Framework for Guiding Funding Decisions about Proposed Research Involving Enhanced Potential Pandemic Pathogens," which implements the OSTP P3CO Framework and lifts the moratorium on funding gain-of-function. (US DHHS, 2017)

July 2018: Follow-up on GOF research. Two research projects that were paused in 2014 are reviewed and approved under the new P3CO policy, allowing them to continue. These projects conclude in 2019. (Imperial et al. 2018; Koblentz and Cassagrande, 2023)

January 23-24, 2020: NSABB meets to discuss transparency of GOF. NSABB held this meeting to discuss the policies and increased transparency related to gain-of-function research and other biosecurity concerns. The meeting

also addressed the board's charge; however, the onset of the COVID-19 pandemic ultimately halted the progress of this version of the NSABB’s charge. (National Institutes of Health, 2020)

March 11, 2020: COVID-19 declared a pandemic: The World Health Organization (WHO) declares COVID-19 a pandemic, which refocuses attention on GOF research, particularly regarding NIH-funded projects involving bat coronaviruses in collaboration with the Wuhan Institute of Virology. (Congressional Research Service, 2022; Cohen, 2021)

March 2021: Renewed scrutiny and calls for transparency. Increased calls for transparency in GOF research and its potential link to the origins of COVID-19 lead to congressional hearings and international discussions on biosafety and biosecurity protocols. (U.S. House of Representatives, 2021)

October 2022: National Biodefense Strategy and Implementation Plan. The White House publishes the National Biodefense Strategy and Implementation Plan, outlining a comprehensive approach to biodefense that includes goals for improving biosafety and biosecurity measures (U.S. White House, 2022)

January 2023: NSABB meets to review draft report recommending updates to DURC and P3CO policies. The National Science Advisory Board for Biosecurity (NSABB) had been charged with reviewing the dual-use research of concern (DURC) policies and the P3CO guidance to evaluate the balance between security and transparency in sharing PPP research information. (NSABB, 2023)

September 13, 2023. Report on the Impact of Research on Infectious Agents. The American Society for Microbiology issues a report titled, “Impact Assessment of Research on Infectious Agents” that the discusses importance of infectious agent research for public health. However, it also addresses concerns around GOF studies. The report recommends standardizing research terminology, increasing transparency and public engagement on the risks and benefits, and strengthening biosafety and biosecurity management systems.

October 2023: HHS updates screening framework for providers and users of synthetic nucleic acids. The Department of Health and Human Services (HHS) releases updated guidelines to improve the screening and oversight of synthetic nucleic acids, aiming to prevent misuse and enhance biosecurity. (US DHHS, 2023)

February 2024: Bulletin of the Atomic Scientists issues report. The Bulletin of the Atomic Scientists publishes "A Framework for Tomorrow’s Pathogen Research: Final Report," compiled by an independent task force of international experts to evaluate the evolving landscape of pathogen research, biosafety, and biosecurity. The report provides key recommendations for managing pandemic risks, advocating for high-probability benefits for public health, the use of less-risky alternatives when possible, and equitable benefit-sharing. It also highlights the importance of trust-building and sustainable governance in pathogen research. (Gupta et al., 2024)

May 6, 2024: OSTP issues new policy on the oversight of DURC/PEPP. The Office of Science and Technology Policy (OSTP) releases a new policy on the oversight of Dual Use Research of Concern (DURC) and Potentially Emerging Pandemic Pathogens (PEPP), emphasizing a comprehensive risk management approach. The new policy consolidates and supersedes the 2012 USG policy for oversight of life sciences dual use research of concern, the 2015 USG policy for institutional oversight of life sciences dual use research of concern, and the 2017 OSTP recommended policy guidance for departmental development of review mechanisms for potential pandemic pathogen care and oversight" (P3CO) (Office of Science and Technology Policy, 2024)

Timeline References

ABSA International. History of ABSA. Mundelein, IL: ABSA International, 1984.

American Society for Microbiology. Impact Assessment of Research on Infectious Agents. American Society for Microbiology, 2023.

Atlas, Ronald, Philip Campbell, Nicholas R. Cozzarelli, Gregory Curfman, Lynn Enquist, Gerald Fink, Annette Flanagin, et al. "Statement on Scientific Publication and Security." Science 299, no. 5610 (2003): 1149.

Berg, Paul, David Baltimore, Hamilton O. Smith, Sydney Brenner, Richard O. Roblin, and Maxine F. Singer. "Summary Statement of the Asilomar Conference on Recombinant DNA Molecules." Proceedings of the National Academy of Sciences 72, no. 6 (1975): 1981-1984.

Centers for Disease Control and Prevention and National Institutes of Health. Biosafety in Microbiological and Biomedical Laboratories (BMBL). 1st ed. Washington, DC: U.S. Government Printing Office, 1984.

Centers for Disease Control and Prevention. "Notice of Proposed Rulemaking (NPRM) to Implement Section 511 of Public Law 104-132, 'The Antiterrorism and Effective Death Penalty Act of 1996'." Department of Health and Human Services, June 10, 1996.

Cohen, Jon. "NIH's Mixed Messages on Controversial Research Spark Confusion." Science 371, no. 6536 (2021): 552-553.

Congressional Research Service. Global Pandemics: Gain-of-Function Research of Concern. 2022.

Federal Experts Security Advisory Panel. Recommendations for the Evaluation and Oversight of Gain-of-Function Research. Washington, DC: Federal Experts Security Advisory Panel, December 2014.

Gryphon Scientific. Risk and Benefit Analysis of Gain of Function Research: Final Report. April 2016.

Gupta, R., Ameenah Gurib-Fakim, Shahid Jameel, Arturo Casadevall, Jesse Bloom, Filippa Lentzos, Aditya Agrawal, Nader AL-Hmoud, Françoise Baylis, Agnes Binagwaho, Sylvie Briand, Rocco Casagrande, Alice Chan, Guang Gao, Andrea George, David Heymann, Claire Jolly, Timothy Kariuki, Jens Kuhn, and Weiqi Zhang. "A Framework for Tomorrow's Pathogen Research: Final Report of the Independent Task Force on Research with Pandemic Risks." 2024.

Herfst, Sander, Eefje JA Schrauwen, Martin Linster, Salin Chutinimitkul, Emmie De Wit, Vincent J. Munster, Erin M. Sorrell et al. "Airborne transmission of influenza A/H5N1 virus between ferrets." science 336, no. 6088 (2012): 1534-1541.

Imperiale, Michael J., Don Howard, and Arturo Casadevall. "The Silver Lining in Gain-of-Function Experiments with Pathogens of Pandemic Potential." In Influenza Virus: Methods and Protocols, edited by Kevin M. Coombs and Suzanne E. LeBlanc, 575-587. New York: Humana Press, 2018.

Jaffe, Howard, Ann P. Patterson, and Nicole Lurie. "Extra Oversight for H7N9 Experiments." Science 341, no. 6147 (2013): 713-714.

Koblentz, Gregory D., and Rocco Casagrande. "Beyond Gain of Function: Strengthening Oversight of Research with Potential Pandemic Pathogens." Pathogens and Global Health (2023): 1-12.

Lipsitch, Marc, and Thomas V. Inglesby. "Moratorium on Research Intended to Create Novel Potential Pandemic Pathogens." mBio5, no. 6 (2014): e02366-14.

National Academies of Sciences, Engineering, and Medicine. Gain-of-Function Research: A Symposium, December 15-16, 2014. Washington, DC: The National Academies Press, 2014.

National Academies of Sciences, Engineering, and Medicine. Gain-of-Function Research: Summary of the Second Symposium, March 10-11, 2016. Washington, DC: The National Academies Press, 2016.

National Institutes of Health. "Establishment of the Recombinant DNA Advisory Committee (RAC)." Federal Register39, no. 10 (1974): 4311-4313.

National Institutes of Health. "National Science Advisory Board for Biosecurity Meeting, January 23-24, 2020. Meeting Summary." Washington, DC: NIH, 2020.

National Institutes of Health. NIH Guidelines for Research Involving Synthetic and Recombinant Nucleic Acid Molecules. Washington, DC: NIH, 1976.

National Research Council. Biotechnology Research in an Age of Terrorism. Washington, DC: The National Academies Press, 2004.
National Science Advisory Board for Biosecurity (NSABB). Proposed Framework for the Oversight of Dual Use Life Sciences Research: Strategies for Minimizing the Potential Misuse of Research Information. Bethesda, MD: National Institutes of Health, Office of Science Policy, May 2015.
National Science Advisory Board for Biosecurity. "Findings and Recommendations of the NSABB Regarding the March 2012 Review of H5N1 Research." Washington, DC: NSABB, 2012.
National Science Advisory Board for Biosecurity. Proposed Biosecurity Oversight Framework for the Future of Science. 2023.
National Science Advisory Board for Biosecurity. Proposed Framework for the Oversight of Dual Use Life Sciences Research: Strategies for Minimizing the Potential Misuse of Research Information. Washington, DC: NSABB, June 2007.
National Science Advisory Board for Biosecurity. Recommendations for the Evaluation and Oversight of Proposed Gain-of-Function Research. Washington, DC: NSABB, May 2016.
Office of Science and Technology Policy. Policy for the Oversight of Dual Use Research of Concern (DURC) and Potentially Emerging Pandemic Pathogens (PEPP). Washington, DC: OSTP, May 6, 2024.
Office of Science and Technology Policy. Recommended Policy Guidance for Departmental Development of Review Mechanisms for Potential Pandemic Pathogen Care and Oversight (P3CO). Washington, DC: OSTP, 2017.
Office of Science and Technology Policy. United States Government Policy for Institutional Oversight of Life Sciences Dual Use Research of Concern (DURC). Office of the Assistant Secretary for Preparedness and Response, 2014.
Sharples, Frances E., John D. Husbands, Anne-Marie Mazza, Audrey Thevenon, and Irene Hook-Barnard. 2015. Potential Risks and Benefits of Gain-of-Function Research: Summary of a Workshop. Washington, DC: National Academies Press.
Torok, T.J., R.V. Tauxe, R.P. Wise, J.R. Livengood, R. Sokolow, S. Mauvais, K.A. Birkness, M.R. Skeels, J.M. Horan, and L.R. Foster. "A Large Community Outbreak of Salmonellosis Caused by Intentional Contamination of Restaurant Salad Bars." JAMA 278, no. 5 (1997): 389-395.
U.S. Congress. Public Law 104-132: Antiterrorism and Effective Death Penalty Act of 1996. U.S. Statutes at Large 110 (1996): 1214-1319.
U.S. Congress. Public Law 107-188: Public Health Security and Bioterrorism Preparedness and Response Act of 2002.U.S. Statutes at Large 116 (2002): 594-626.
U.S. Department of Health and Human Services. "A Framework for Guiding U.S. Department of Health and Human Services Funding Decisions About Research Proposals with the Potential for Generating Highly Pathogenic Avian Influenza H5N1 Viruses That Are Transmissible Among Mammals by Respiratory Droplets." February 21, 2013.
U.S. Department of Health and Human Services. "Additional Requirements for Facilities Transferring or Receiving Select Agents." Federal Register 61, no. 207 (1996): 55190-55202.
U.S. Department of Health and Human Services. “Framework for Guiding Funding Decisions about Proposed Research Involving Enhanced Potential Pandemic Pathogens (P3CO).” 2017
U.S. Department of Health and Human Services. “Guidance for Funding Proposals Involving HPAI H5N1 Research That May Be Dual Use Research of Concern (DURC).” February 2013.
U.S. Department of Health and Human Services. "HHS Announces Formation of a Trans-Federal Task Force on Optimizing Biosafety and Biocontainment Oversight." October 4, 2007.
U.S. Department of Health and Human Services. “National Science Advisory Board for Biosecurity Inaugural Meeting Summary.” July 2005.
U.S. Department of Health and Human Services. "News Release: HHS Names Select Agent Advisory Committee." March 4, 2004.

U.S. Department of Health and Human Services. "Possession, Use, and Transfer of Select Agents and Toxins." Federal Register, December 12, 2002.
U.S. Department of Health and Human Services. "Screening Framework Guidance for Providers of Synthetic Double-Stranded DNA." October 13, 2010.
U.S. Department of Health and Human Services. "Updated Screening Framework Guidance for Providers and Users of Synthetic Nucleic Acids." October 2023.
U.S. Government Accountability Office. “Bioterrorism: Public Health Response to Anthrax Incidents of 2001.” Washington, DC: U.S. Government Accountability Office, 2003.
U.S. Government. "United States Government Gain-of-Function Deliberative Process and Research Funding Pause on Selected Gain-of-Function Research Involving Influenza, MERS, and SARS Viruses." Office of Science and Technology Policy, October 17, 2014.
U.S. Government. “United States Government Policy for Oversight of Life Sciences Dual Use Research of Concern.” March 2012.
U.S. House of Representatives. "Hearing on the Origin of COVID-19: An Examination of the Possible Sources and the Path Forward." 2021.
U.S. White House. "Executive Order 13486: Strengthening Laboratory Biosecurity." January 9, 2009.
U.S. White House. "Executive Order 13546: Optimizing the Security of Biological Select Agents and Toxins in the United States." July 2, 2010.
U.S. White House. National Biodefense Strategy and Implementation Plan. Washington, DC: U.S. Government, October 2022.
USA PATRIOT Act, Public Law 107-56, 107th Congress. 2001.
Wivel, Nelson A. "Historical Perspectives Pertaining to the NIH Recombinant DNA Advisory Committee." Human Gene Therapy25, no. 1 (2014): 19-24.
World Health Organization. "WHA58.29 Enhancement of Laboratory Biosafety." World Health Assembly, May 25, 2005.
World Health Organization. Laboratory Biosafety Manual. 1st ed. Geneva: WHO, 1983.
